# Distinctive Features of NREM Parasomnia Behaviors in Parkinson’s Disease and Multiple System Atrophy

**DOI:** 10.1371/journal.pone.0120973

**Published:** 2015-03-10

**Authors:** Pietro-Luca Ratti, Maria Sierra-Peña, Raffaele Manni, Marion Simonetta-Moreau, Julien Bastin, Harrison Mace, Olivier Rascol, Olivier David

**Affiliations:** 1 Department of Clinical Pharmacology, Toulouse University Hospital and Paul Sabatier University, Toulouse, France; 2 UMR 825 Brain imaging and neurological dysfunctions, INSERM, Toulouse, France; 3 Sleep and Epilepsy center, Neurocenter of Southern Switzerland, Civic Hospital (EOC) of Lugano, Lugano, Switzerland; 4 Department of Neurology, Toulouse University Hospital and Paul Sabatier University, Toulouse, France; 5 Sleep Medicine and Epilepsy unit, C. Mondino National Neurological Institute, Pavia, Italy; 6 Brain Function & Neuromodulation, Grenoble Institute of Neuroscience, Grenoble, France; 7 Joseph Fourier University, Grenoble, France; 8 Reference Centre for Multiple System Atrophy, Toulouse University Hospital, Toulouse, France; University of Oxford, UNITED KINGDOM

## Abstract

**Objective:**

To characterize parasomnia behaviors on arousal from NREM sleep in Parkinson’s Disease (PD) and Multiple System Atrophy (MSA).

**Methods:**

From 30 patients with PD, Dementia with Lewy Bodies/Dementia associated with PD, or MSA undergoing nocturnal video-polysomnography for presumed dream enactment behavior, we were able to select 2 PD and 2 MSA patients featuring NREM Parasomnia Behviors (NPBs). We identified episodes during which the subjects seemed to enact dreams or presumed dream-like mentation (NPB arousals) versus episodes with physiological movements (no-NPB arousals). A time-frequency analysis (Morlet Wavelet Transform) of the scalp EEG signals around each NPB and no- NPB arousal onset was performed, and the amplitudes of the spectral frequencies were compared between NPB and no-NPB arousals.

**Results:**

19 NPBs were identified, 12 of which consisting of ‘elementary’ NPBs while 7 resembling confusional arousals. With quantitative EEG analysis, we found an amplitude reduction in the 5-6 Hz band 40 seconds before NPBs arousal as compared to no-NPB arousals at F4 and C4 derivations (p<0.01).

**Conclusions:**

Many PD and MSA patients feature various NREM sleep-related behaviors, with clinical and electrophysiological differences and similarities with arousal parasomnias in the general population.

**Significance:**

This study help bring to attention an overlooked phenomenon in neurodegenerative diseases.

## Introduction

Patients affected with alpha-synucleinopathies (Parkinson’s Disease – PD, Dementia with Lewy Bodies – DLB/Dementia associated with Parkinson’s Disease – PDD, or Multiple System Atrophy – MSA) and their bed-partners frequently seek medical attention for “abnormal” sleep-related events that can consist of movements or behaviors of different complexity. Video-polysomnography (PSG) recordings demonstrate that these parasomnia behaviors observed in patients with synucleinopathies (which were elsewhere referred to as “paroxysmal behaviors” [1–3] or “sleep enactement behaviors” [4]) consist of heterogeneous behaviors, encompassing REM behavior disorder (RBD) as well as Arousal-related Motor-Behavioral Episodes (AMBEs) emerging at arousal from NREM or REM sleep [1–4]. In all of these parasomnia behaviors, the patients seem to act out a dream (as in the case for RBD or pseudo-RBD [1,5]) or a dream-like mentation occurring in intermediate states between sleep and wakefulness [1,2,6,7]. The occurrence of NREM parasomnias resembling sleepwalking or confusional arousals has only been recently reported in PD and DLB/PDD [1,8–10], but it represents a still overlooked phenomenon in alpha-synucleinopathies. Moreover, a well-defined clinical and electrophysiological characterization is still lacking. This preliminary study is aimed at further characterizing NREM Parasomnia Behaviors (NPBs) in alpha-synucleinopathies through a video-PSG analysis of highly selected events and a quantitative EEG correlate using a Morlet Wavelet Transform method.

## Methods

### Setting and patients’ selection

Thirty consecutive patients with an alpha-synucleinopathy (10 idiopathic PD not treated with deep brain stimulation, 8 probable or possible PDD or DLB, and 12 probable or possible MSA [11–14]) were referred to the third-level Sleep laboratory for Movement Disorders of Toulouse University Hospital between January 2010 and December 2011. These patients’ report of dream enactment behaviors (“rêves agis”) was confirmed by a bed-partner and not yet investigated by means of PSG; all of the subjects underwent a video-PSG between June 2010 and December 2011. A retrospective analysis of their video-PSG recordings was then performed.

Motor impairment was assessed by the Unified Parkinson’s Disease Rating Scale – part III [11] or by the Unified Multiple System Atrophy Rating Scale – part II [15] in PD, DLB/PDD and in MSA patients, respectively. All of the patients were examined for their sleep complaints by a neurologist expert in sleep medicine (PLR). The Epworth Sleepiness Scale [16] was also administered. Deep brain stimulation was an exclusion criterion for this study.

### Ethics statement

All patients gave written informed consent after full explanation of the procedure. This study was approved by the ethics committee and review board of Toulouse University Hospital (authorization n. 36-0414).

### Video-polysomnography

Subjects underwent a full-night video-PSG recording. The recordings were acquired by a Grass AS-40 Plus system and the Twin software version 4.5 (Grass Technologies Inc., West Warwick, RI, U.S.A.).

The PSG montage included: scalp EEG with electrodes positioned at F3; C3; O1; F4; C4; O2, Fz; Cz; Pz; A1; A2, in accordance with the International 10–20 System, referring to a common electrode in CPz; an electro-oculogram, a surface electromyogram of the chin, the flexor digitorum superficialis (upper limbs), and the extensor digitorum brevis (lower limbs) muscles bilaterally [17–19]. Finally, the PSG montage incorporated airflow (nasal cannula), snoring sounds (microphone), measurement of thoracic respiratory effort (piezoelectric strain gauges), and arterial oxygen saturation level (pulse oximeter with finger probe). Synchronised digital infrared video tracks and ambient sound recordings were obtained in order to perform a simultaneous analysis of PSG track and sleep-related behaviors. The patient’s usual sleep and daily living routines, as well as medications, were kept unchanged during the two weeks prior to the clinical and instrumental assessment.

Visual analysis of the PSG recordings was performed by a trained sleep scorer (PLR) according to standard criteria [20], taking into account previously published recommendations and suggestions for sleep scoring in PD. The following two criteria determined the selection of the patients’ recordings for further analyses: a) Recognition of REM and NREM sleep patterns at video-PSG; b) Identification of parasomnia behaviors upon arousals from NREM sleep.

### Detection and selection of NREM arousals with and without parasomnia behaviors

All the NREM arousals in each of the selected recordings were visually inspected. The arousal onset was determined on the basis of the EEG as the sudden onset of alpha and/or desynchronized activity lasting at least 3 seconds [20]. Only arousals preceded by at least 2 minutes of stable NREM sleep (i.e. AASM stage N1, N2, or N3, or for the recordings in which AASM criteria were not applicable, NREM sleep [21] without arousals) and at least 10 minutes before or after an epoch of REM sleep were considered. In order to exclude arousals at NREM-REM sleep transitions, we also excluded NPB arousals within 10 minutes of an epoch with a marked reduction of chin muscular tone and disappearance of K complexes or sleep spindles, even in the absence of rapid eye movements.

The video recording corresponding to each NREM arousal was visually inspected by an expert sleep scorer (PLR), for each video-PSG recording; all the movements or behaviors accompanying each arousal were systematically examined. Arousals accompanied by single jerks of one or more body segments were not considered, as the specificity of their categorization, pathophysiology, and significance might be questionable. In fact, they might represent either normal physiologic findings or, on the other hand, the expression of motor control impairment during sleep associated with neurodegeneration.

All the final selected NREM arousals were classified either as “NPB arousals” or “no-NPB arousals”. NPB arousals were identified on the basis of their clinical features on the video recordings [1,4]. No-NPB arousals were defined as arousals associated with a two-fold increase in chin muscular tone (as compared to the previous epoch) and either associated with movements indicative of postural changes or not associated with movements on the video. Among all the no-NPB arousals, we chose to select only no-NPB arousals associated with increased chin EMG activity; this was standardized in order to differentiate the EEG changes between NPB arousals and no-NPB arousals and not simply due to cortical activation preceding movements.

### Computation of the EEG spectral power for NPB and no-NPB arousals

The same procedure was applied to quantify the EEG spectral components at F3, C3, O1, F4, C4, O2, Fz, Cz, Pz derivations (referred to as A1+A2) for NPB and no-NPB arousals.

First, we selected a 3-minute EEG extract around each NREM arousal onset; this extract began120 seconds before and ended 60 seconds after each arousal onset. The subsequent statistical analysis was performed using the Statistical Parametric Mapping software (SPM8, Wellcome Department of Imaging Neuroscience, www.fil.ion.ucl.ac.uk/spm). For each extract, the scalp EEG signals were transformed into the time-frequency plane using a Morlet Wavelet Transform. The EEG amplitude A(*t*,*f*) at time *t* and frequency *f* was defined as the amplitude of the Morlet Wavelet Transform in the frequency range [1 30] Hz (frequency resolution = 0.25 Hz) and time range [−60 10] s (time resolution = 50 ms). The EEG amplitude of each derivation was z-scored according to a baseline Morlet Wavelet Transform. The baseline was constructed for each patient by concatenating the baseline Morlet Wavelet Transform of every event, which selected a 30-s duration corresponding to an artefact and arousal-free EEG window 20–120 seconds before the NPB or no-NPB arousal.

For each derivation, we thus obtained a measure of normalised EEG amplitude A(*t*, *f*) at time *t* and frequency *f* for NPB and no-NPB arousals. Finally, we smoothed the images of normalised amplitude (full–width, half-maximum of filter: 2 Hz – 10 s) and performed a two-sample t-test, based on a fixed-effect analysis among patients, in order to detect differences in EEG amplitude between NPB and no-NPB arousals.

### NREM Parasomnia Behaviors classification according to motor pattern

All the video clips were exported from the PSG recordings (with no time reference) and anonymized. They were randomized and independently shown to a neurologist expert in movement disorders (MSP), which was blind on any additional information on the patients PSG recordings, and to a neurologist expert in sleep medicine (PLR). NPB events were then independently classified by each scorer on the basis of their video features as:

1. Elementary NPBs, consisting of either isolated behaviors (e.g. smiling, laughing, talking) or seemingly purposeless, composite movements (e.g. combination of different movements like jerks, slow raise of an arm or of a leg, head turning);
2. Confusional NPBs: head-orienting response associated with slow movements, which may or may not be accompanied by trunk raising or vocalisations, as if calling out to someone.

### Video characterisation of the motor pattern of NREM Parasomnia Behaviors

The video recordings corresponding to NPBs were carefully and independently described by the two scorers (MSP and PLR) before being revised together. In the event of discrepant descriptions, an agreement on a definite description was reached by again revising the videos. A native English-speaking fellow in bioengineering (HM) independently revised each video recording and checked the description. An agreement among the three scorers was obtained for doubtful cases after additional video analysis.

The following parameters were evaluated for each NPB event: sleep stage preceding the arousal, timing of the arousal from sleep onset, triggering factors, and event duration. The end of each event was defined either as the end of any movement or the moment in which the subject laid back to bed—or (in one case) by the entrance of the sleep technician in the room to assist the patient. In addition, for each event including NPBs, the following neurological motor signs were also systematically assessed: hypokinesia, bradykinesia, and tremor.

## Results

Twelve patients (4 PD, 6 DLB/PDD and 2 MSA) of the 30 screened were excluded because distinct patterns of NREM and REM sleep were not recognizable; with REM sleep undetectable, it was judged doubtful that covert REM sleep may have been underlying some NREM sleep periods.

Parasomnia behaviors on arousals from NREM sleep were identified in 6 patients (3 PD, 3 MSA) of the 18 remaining patients. After selecting parasomnia behaviors at least 10 minutes distant from REM sleep or muscle atonia among all these events, two other patients were excluded from further analyses.

**Fig 1:**
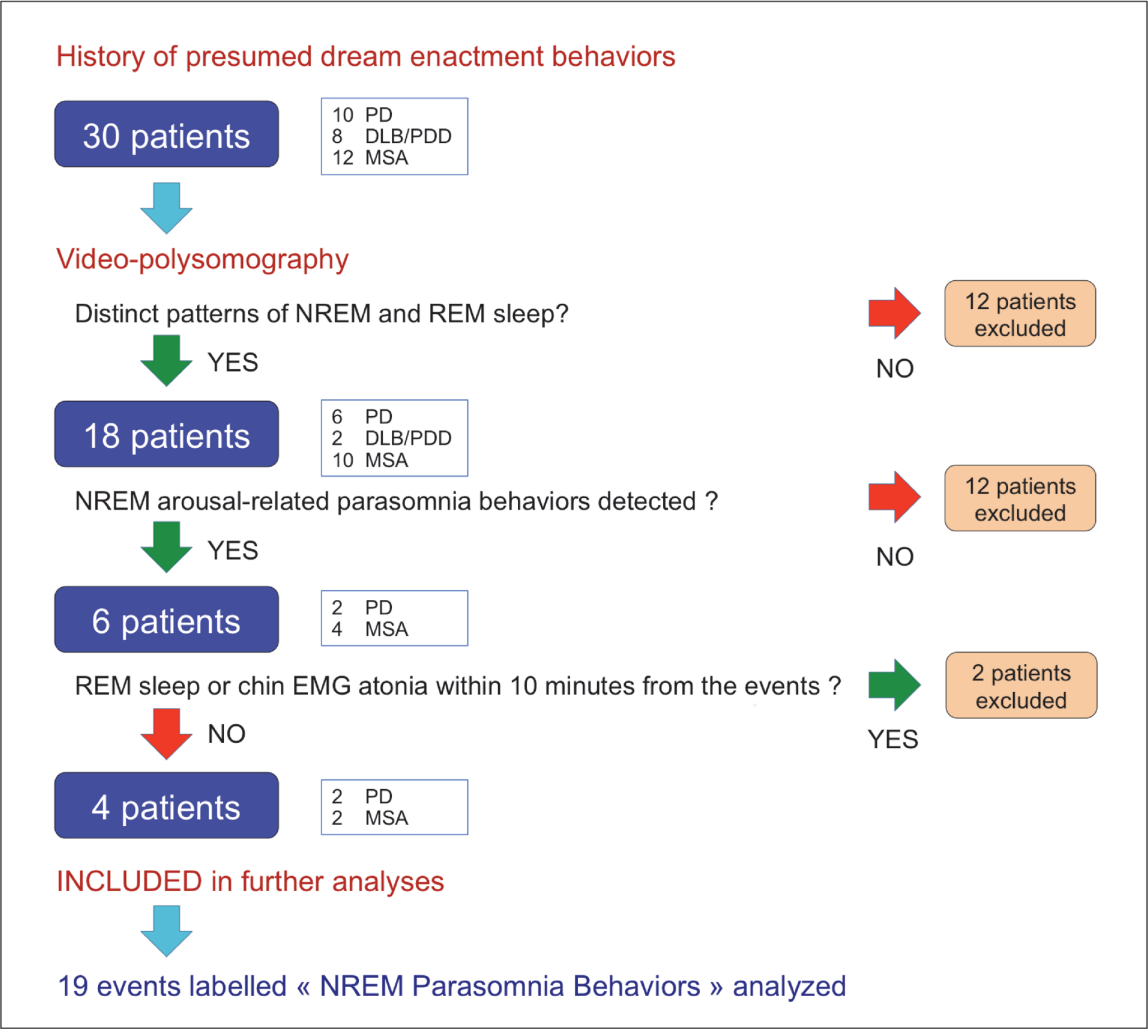
Flow chart explaining the selection procedure of the patients and on the NREM Parasomnia Behaviors (NPBs) on which subsequent analyses were conducted.

At the end of this selection procedure, 19 parasomnia behaviors on arousals from NREM sleep were identified from the recordings of four of the thirty screened patients (2 PD and 2 MSA). We refer to these strictly selected behavioral events as to “NPBs”. Fig. 1 summarizes the selection procedure of the patients and the NPB events on which the final analyses were conducted. Two PSGs were recorded for Patient n°1, as this individual was participating in a non-pharmacologic clinical study.

The demographic and clinical features of the 4 selected patients are summarized in Table 1.

No patient reported sleep terrors, or sleepwalking or confusional arousals in childhood or before PD/MSA onset. All four patients exhibited concomitant REM behavior disorder, so they also had parasomnia overlap disorder [22]. All the four patients had sleep-disorder breathing, which was severe in one case, moderate in another, and mild in the other two patients. Both the patients with MSA had a high periodic limb movement index, and only one of them (patient 3) had symptomatic restless legs syndrome.

**Table 1:** Demographic and clinical features of the four patients studied.

	Patient n°				
	1		2	3	4
Sex	M		M	M	M
Diagnosis	PD		PD	MSA-P	MSA-C
Age	77.3		69.3	71.3	61.9
PD/MSA duration (years)	22		8	5	2
UPDRS-III	27		63		
UMSAR-II				20	17
Antiparkinsonian drugs	L-DOPA DA		L-DOPA DA COMT-I	L-DOPA	
L-DOPA daily equivalent dose (mg)	840		1848	800	0
Psychotropic drugs			MLT		SRI
Epworth Sleepiness Scale	12		15	16	16
Presence of visual hallucinations	no		yes	no	no
Polysomnographic parameters	PSG1	PSG2			
Dominant Occipital Frequency (Hz)	6	6	7	12	11
Total Sleep Time (min)	430	434	272	316	367
Sleep Efficiency (%)	76.3	78.5	54.1	82.7	83.4
Non REM sleep (%)	95.7	95.4	94.3	93.6	90.6
N1 (%)			8.1	43.9	25.6
N2 (%)			77.4	46	47.8
N3 (%)			8.8	1.7	17.2
R (%)	4.3	4.6	5.7	8.4	9.4
WASO/TIB (%)	46.5	18.3	48.3	17.2	16.0
Arousal index	15.1	18.8	13.2	37.8	22.4
Limb movement in sleep index	0.0	0.0	0.0	3.0	2.8
Periodic limb movements in sleep index	0.0	0.0	0.0	161.0	50.3
Respiratory Disturbance Index	24.4	14.9	1.9	60.3	NA
Oxygen Desaturation Index ≥4%	16.6	7.0	8.6	64.1	5.7

WASO = Wake After Sleep Onset

TIB = Time in Bed

Patient n°1 had two video-PSG recordings as a part on a clinical study. N1, N2 and N3 NREM sleep stages were not recognizable in this patient, although REM and NREM sleep patterns were still recognizable.

### Video analysis of NPB events

NPBs were classified as follows: twelve episodes of elementary NPBs and seven confusional-type NPBs. The two scorers independently assigned all the episodes to one of these categories with complete concordance (Cohen’s k-index = 1). Table 2 provides a description of the video features of each of the nineteen NPB events and of its polysomnographic correlates.

**Table 2:** Video Characteristics of the NREM Parasomnia Behaviors.

**Elementary NREM Parasomnia Behaviors**									
**Patient and diagnosis**	**Ep**	**TSO (min)**	**Sleep stage**	**Trigger**	**Event description**	**Dur (s)**	**Motor signs**		
							**T**	**A**	**B**
1A PD	1	170	NREM	Snoring	Incomprehensible words as if calling someone, eyes open, flexion of both legs and feet, slow movements of the right hand touching nose.	40	+	+	+
1B PD	2	68	NREM	Apnea	Sequential repeated rapid jerks of the head, left and right upper limb, and right lower limb.	3	NA	NA	−
1B PD	3	100	NREM	RERA	Brisk abduction of both upper limbs followed by composite rapid movements of right upper limb and both lower limbs.	4	−	NA	NA
1B PD	4	180	NREM	Apnea	Sudden rapid movements of the four limbs simultaneously, slow movements of the upper limbs under the blankets and whispering incomprehensible words.	19	+	+	NA
3 MSA-P	5	97	N2	Apnea	Four quick flexions of the left knee resulting in elevation from the bed; followed by three quick, asynchronous flexions and extensions of the feet.	5	−	NA	−
3 MSA-P	6	125	N3	Apnea	Unhurried flexion of the right knee; followed by brisk flexion of the right knee, then of the right hip, knee, and foot; accompanied by a brisk rising of both arms and a light, swift rotation of the head to the right.	4	−	−	−
3 MSA-P	7	176	N1	Apnea	Unhurried flexion of the right knee; followed by sudden flexion and extreme elevation of the right leg; followed by a brief inspiration and a small jerk in both arms.	6	−	−	−
3 MSA-P	8	187	N2	Apnea	Sudden flexion of both legs, accompanied by a quick shaking of the left hand	3	−	−	−
4 MSA-C	9	58	N2	RERA	Jerky head extension followed by a jerk of the upper limbs and of the right lower limb.	3	NA	NA	NA
4 MSA-C	10	89	N3	No	Repeated quick movements of the left upper limb under the blankets, accompanied by multiple turns and raisings of the head and shoulders in both directions.	6	+	NA	−
4 MSA-C	11	237	N1	No	Small jerky head extension followed by jerks of the left lower limb and a small movement of the right hand under the blanket.	6	+	NA	NA
4 MSA-C	12	419	N1	No	Sudden elevation of the left upper limb.	1	−	NA	−
**Confusional NREM Parasomnia Behaviors**									
**Patient diagnosis**	**Ep**	**TSO (min)**	**Sleep stage**	**Trigger**	**Event description**	**Dur (s)**	**Motor signs**		
							**T**	**A**	**B**
1A PD	13	36	NREM	Apnea	Groan, eyes open, accompanied by a slow turn of the head to the left and then to the right, followed by head raising and vocalisation with gesture of the right hand, as if calling somebody. Looking around several times with small movements of both hands before lying back down to sleep.	33	−	+	−
1A PD	14	61	NREM	Apnea	Eyes open, incomprehensible words, accompanied by looking ahead and around, with slow, repeated head raisings and turnings to both sides. Episode ends with final head raising.	49	−	+	NA
1A PD	15	84	NREM	Apnea	Eyes open, slow head and trunk raising with flexion of the legs as if attempting to sit, speaking incomprehensible words to unreal people.Slow head turning to the left, continued speech and leg flexions, head raising and repeated attempts to sit on the bed with trunk raising; then uncovering of the bedsheets and a return to lying in the bed. Prolonged rubbing of eyes with left arm.	82	−	+	−
1A PD	16	165	NREM	RERA	Incomprehensible whispered words accompanied by slight head movements.	12	−	NA	NA
1A PD	17	179	NREM	Apnea	Vocalisation, slight raising of the head, iterative vocalisations and head raising as if looking/calling for someone, then lying back down and closing eyes.	41	−	+	NA
1B PD	18	142	NREM	Apnea	Head raising, sitting upright on the edge of the bed and removing the blankets (all movements rapid and jerky), looking around several times and touching chest with the left hand. After an attempt to lie back down in the bed under the covers (the nurse enters and the patient explains to her that he has been dreaming and he had misinterpreted his dreams for reality).	49	+	−	−
2 PD	19	60	N3	LM	Incomprehensible whispered words, slight turn of the head to the right, head raising and eyes opening, looking around slowly. Then, several attempts to remove (with eventual success) his elastic net bandage–cap; removal of some electrodes with both hands, all while continuing an incomprehensible speech. When the sleep technician enters the room, the patient turns to the door upon entry; patient tells nurse that he thought he was at home and that he had seen his granddaughter.	75	−	+	+

A: Akinesia

B: Bradykinesia

Dur: Event duration

Ep: Episode number

LM: Limb Movement

MSA-C: Multiple system atrophy, cerebellar-type

MSA-P: Multiple system atrophy, parkinsonian-type

NA: Not Assessable

PD: Parkinson’s Disease

RERA: Respiratory Event-Related Arousal

T: Tremor

TSO: Time from Sleep Onset

+: Motor sign present

−: Motor sign absent

*: Trigger not assessable as the respiratory channels were not functioning

All the confusional NPBs occurred either within the first hour and half or between two and three hours after the onset of sleep, while elementary NPBs were more variably distributed across the entire sleep period.

We observed the confusional-type NPBs only in PD patients, while both PD and MSA patients exhibited elementary NPBs. Although the limited number of patients and events prevent larger generalizations, we did not notice overt semiological differences between elementary NPBs occurring in patients with PD and MSA. Similarly, we did not notice inter-individual differences in the expression of elementary- or confusional-type NPBs. Elementary NPBs were shorter in duration than confusional-type NPBs (5.5 ± 4.8 s vs. 48.7 ± 24.0 s, respectively). In all of the confusional-type NPBs, the patients seemed to interact with unreal people or objects. In two of these episodes (in two different patients), the sleep technician entered the room and recorded the patient’s recall: in both cases, the patients referred to have being dreaming and interacting with their dream scenarios, which consisted of people watching them.

Elementary NPBs consisted of slow and/or jerky movements in variable combinations, but different movements within the same episode seemed neither goal-directed nor coordinated. With video analysis, the patients’ movements during confusional NPBs were often labelled as bradykinetic or hypokinetic (in 5 out of the 6 confusional NPBs in which this feature was assessable); this differed from most movements in the set of elementary NPBs, which were labelled normokinetic in seven out of the 9 assessed episodes. Tremor more frequently accompanied elementary NPBs (4 out of the 10 assessable episodes) rather than confusional NPBs (1 out of the 7 episodes). On the other hand, we could not notice any association between the characteristics of the movements of NPBs (jerky/rapid/brisk vs. slow/unhurried) and parkinsonian motor signs.

Of the 19 NPB arousals, 3 emerged from N1 sleep, 3 from N2, 3 from N3 and the remaining 10 (in the patient in which N1, N2 and N3 stages were not easily recognizable) from undifferentiated NREM sleep. Of the 38 no-NPB arousals, 2 emerged from N1 sleep, 18 from N2, 5 from N3, and 13 from undifferentiated NREM sleep. The NREM sleep stage was not associated with the occurrence of NPB vs. no-NPB arousals (p>0.024, not significant after Bonferroni’s correction). Different triggers of the NPB and no-NPB NREM arousals were recognized: respiratory events (apneas, hypopneas, respiratory efforts or snoring) and limb movements, while some arousals appeared spontaneous. The occurrence of a NPB or a no-NPB arousal was not influenced by the type of triggering factor (p>0.195), although the sample is too small to conclude on this point. A respiratory event was recognized to trigger 12 out of the 19 NPB arousals (63.2%) and 14 out of 38 no-NPB arousals (36.8%) (p>0.600). In particular, 5 out of 7 confusional NPBs were preceded by an apnea and one of them by a Respiratory Effort-Related Arousal (RERA). An oxygen saturation drop of 4% was found in 4 out of 19 (21.1%) NPB arousals and 9 out of 38 (23.7%) no-NPB arousals (p>0.663). No significant difference was observed in the pre-arousal oxygen saturation value, oxygen desaturation drop, or oxygen desaturation duration in NPB vs. no-NPB arousals (p>0.619).

### Computation of the pre-arousal EEG frequency spectrum of NPB vs. no-NPB arousals

For the EEG spectral computation, these 19 NPB arousals were compared with no-NPB arousals from the PSG of the same four patients. We identified fifty-nine events fulfilling the selection criteria for no-NPB arousals, described previously.

**Fig 2:**
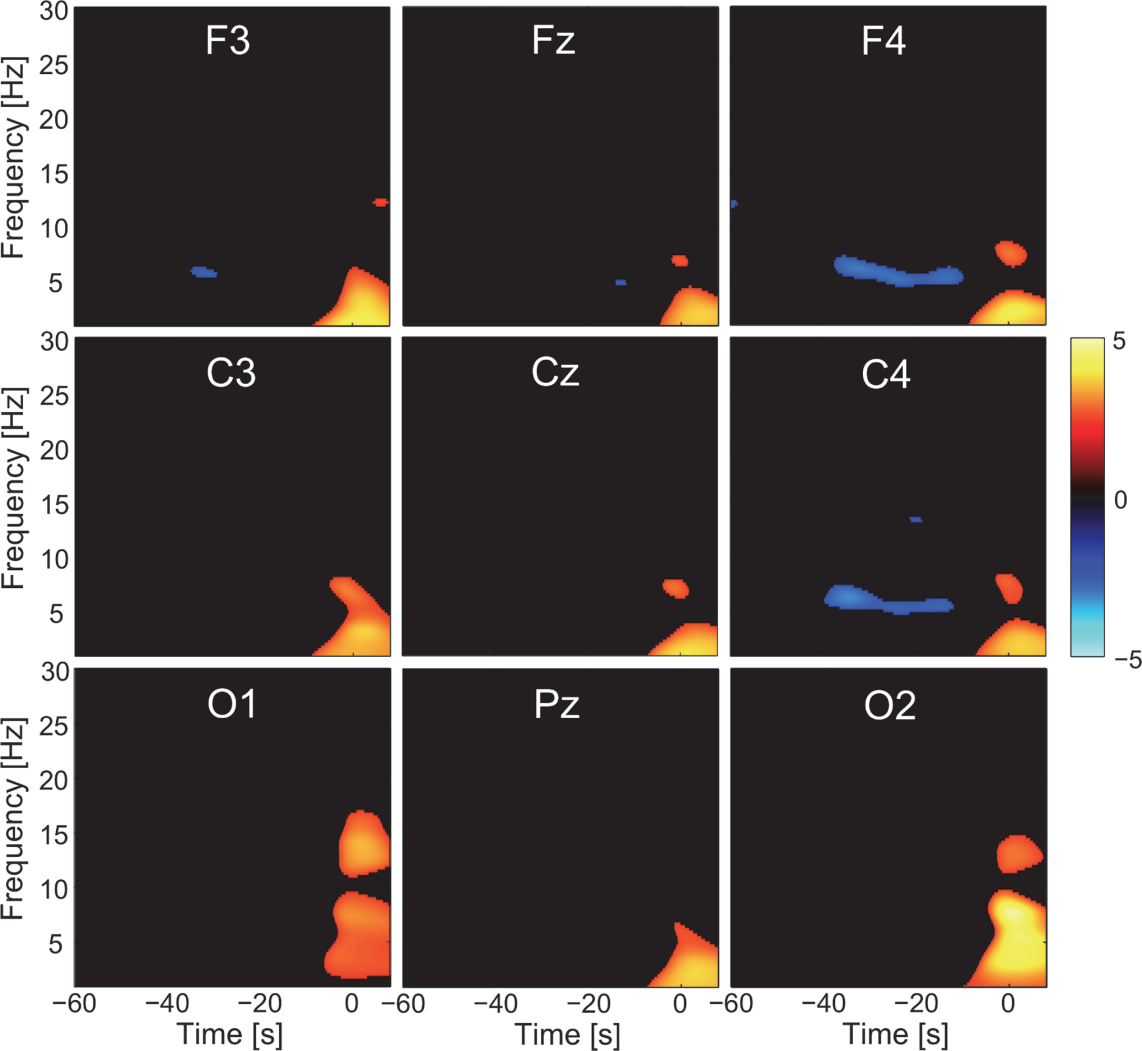
Time–frequency chart of t-statistics comparing normalized EEG amplitude for the NPB arousals versus no-NPB arousals at F3, Fz, F4, C3, Cz, C4, Pz, O1, and O2. The arousal onset is the 0 of the time axis. Note a significant amplitude reduction in the 5–6 Hz range frequency (light blue horizontal line) in NPB compared to no-NPB arousals at F4 and C4 derivations, starting around 40 seconds before the arousal. The orange-and-yellow spot around the arousal is due to movement artefact and is not the expression of an increase in the low frequency band amplitude. The t-map was thresholded for display at p<0.01 uncorrected.

The time-frequency analysis showed a significant amplitude reduction in the 5–6 frequency range in NPB compared to non-NPB arousals at F4 and C4 derivations, starting around 40 seconds before the arousal and ending at the arousal (p<0.01, uncorrected). The movement artefact accompanying the arousal appeared in the time-frequency plot as an amplitude increase in the low frequencies across all derivations. The frequency spectrum of the post-arousal EEG was not calculated due to movement artefacts. Fig. 2 depicts the time–frequency chart of normalized EEG amplitude for the NPB arousals compared to no-NPB arousals.

## Discussion

In this work, we chose to apply very strict selection criteria to precisely identify and describe late-onset parasomnia behaviors unequivocally emerging from NREM sleep arousals (differently from other previous reports [2,4,8,23–26]) in a highly selected group of patients with alpha-synucleinopathies. We named these events NPBs. We report NPBs in two patients with PD and, for the first time, in two patients with MSA.

From a semiological point of view, NPBs encompass seemingly purposeful or purposeless movements and behaviors in different combinations which not always resemble the complex, coordinated behaviors of ‘classical’ disorders of arousal [22]. In none of our cases, the patients showed the classical out-of-the bed behavior of sleepwalking. Similarly to disorders of arousal, in many confusional NPBs the subjects interacted with their environment in an apparently oneiric state.

Some of the behaviors we describe share semiological similarities to parasomnia overlap disorder events [27–30] and from NREM arousal behaviors reported with sleep-disorder breathing in an adult population with previously PSG-confirmed disorder of arousal [31]. Due to our strict selection criteria of the events, we challenge the hypothesis that NPBs could be the behavioral outcome of REM sleep dream mentation upon arousals occurring at transitions or intermediate states between NREM and REM sleep [21,32–34], according to the “covert REM” hypothesis [35]. Moreover, confusional NPBs in particular appeared different from the violent, more frightening events of REM behavior disorder. In this sense, NPBs seem to represent a different entity from pseudo-REM behavior disorder [5]. Finally, NPBs also differ from delirium that might be observed in sleep-disordered breathing [36–39]. In fact, NPBs have an abrupt onset, are self-limiting (always lasting less than 90 seconds) and lack the excitation and violence characterizing delirium.

The semiological features of non-rytmical, non-stereotyped combined movements and behaviors also suggest that NPBs we report here are of cortical origin. Thus, they cannot only be explained as the release of central pattern generators like in disorders of arousal or frontal or temporal nocturnal seizures [40]. The electrophysiological correlates of NPBs at EEG time-frequency analysis also seem to indicate that NPBs might involve cortical networks. However, the finding of an amplitude reduction in the 5–6 Hz (theta) frequency band at right frontal and central cortices preceding NPB arousals seems to point towards a different pathophysiology underlying NPBs as compared to ‘classical’ arousal parasomnias such as sleepwalking and confusional arousals in adult sleepwalkers. The latter are interpreted as dissociative states of consciousness in which the premotor and motor cortices are awake while NREM sleep persists at the associative cortices level, while the ascending arousal system is defective [41,42]. Their electrophysiological correlates (increase in central and parietal delta activity preceding the somnambulistic episodes [43] and persistence of delta activity of N3 sleep intermixed with alpha or beta activity in most post-arousal EEG [44]) also differ from the one of NPBs. Tonic theta activity during wakefulness correlates to higher cognitive performance, attention shift and working memory in healthy subjects [45]. On the other hand, decreased theta activity in wakefulness correlates to sleep inertia [46,47]. We hypothesize NPBs in our patients to reflect a «low performance» state of the awaking cerebral cortex which fails to integrate the information and attention shift from the NREM sleep mentation to the sensory information coming from the environment. The attention shift involves a widespread cortico-subcortical network in which the cingulate gyrus and the medial prefrontal cortex play a major role [48]. The right cerebral hemisphere bears a specialization for the dynamic reallocation of spatial attention within the extrapersonal space [49,50]. We suggest defective information processing due to functional impairment of this network and of the ascending arousal system activity during NPB arousals. We hypothesize that, during NPBs, dysfunctional prefrontal and frontal cortices either fail to get a sensory feedback from the real world, to take control of central pattern generators, or to inhibit NREM sleep mentation upon arousal. A combination of different levels of arousal, awareness, and/or motor inhibition could account for the actual behavioral outcome of the NPBs (elementary- or confusional-type). Different levels of motor inhibition and/or activation of pyramidal and extra-pyramidal motor circuits during the arousals might also explain while the movements observed during the NPBs were variably associated with different expressions of tremor, akinesia or bradykinesia and different combinations of jerky/rapid/brisk and slow/unhurried movements.

Worthy of note, all four patients in our sample were using dopaminergic agents and/or psychotropic medications, raising the hypothesis that the network impairment accounting for NPBs might be due to the combination of structural (i.e. neurodegeneration) and functional (drug-induced) dysfunction. Different triggers elicited similar behaviors and only some out of all NPBs, independent of their triggering factors, were associated with “abnormal” or “regular” behaviors, even in the same patients. The majority of the NPBs, yet not all of them, emerged from arousals triggered by respiratory events. The data presented here are too limited to draw universal conclusions on the relationship between NPBs and their triggers.

The PSG recordings of the four analyzed patients showed impaired sleep macrostructure, with reduced percentage of REM sleep and (when detectable) of N3 sleep, probably as a consequence of sleep instability, as indicated by an increased arousal index or wake after sleep onset time in all the recordings. It could be speculated that this sleep instability might act as predisposing or triggering factor for NPBs to occur.

This study suffers from several limitations. In particular, we were not able to collect the patient’s report in most NPB episodes, and thus to confirm whether the NPBs corresponded in fact to dream or dream-like enactment behavior. Moreover, the study is conducted on a limited number of observations in a small set of patient samples and in the absence of a control group. Finally, after the selection procedure, no patient with DLB was included in the analysis.

## Conclusions

Our results seem to suggest that NPBs might be a rather uncommon, late-onset, neurodegeneration-related phenomena observed in alpha synucleinopathies. In fact, differently from the disorders of arousal observed in the general population, NPBs are observed in late stages of PD or in MSA and they are not a relapse of antecedent NREM parasomnias. If some confusional NPBs could meet the criteria for confusional arousals in the general populations [22], this is not the case for elementary NPBs. For these reasons, we think that NPBs cannot be considered in a strict sense ‘classical’ disorders of arousal. On the other hand, they could be interpreted within the continuum of the intricate pathophysiology of arousal disorders involving dysfunction of cortical and subcortical networks. Future investigations on larger patients’ populations, with more extended EEG mapping and with systematical assessment of subject’s recall of the pre-arousal mentation, could shed more light on the ultimate neurobiological significance of these events. The potential influence of the NREM sleep stage on the occurrence of NPB vs. NPB arousals should also taken into account in future studies.
